# The impact of chronic conditions of care recipients on the labour force participation of informal carers in Australia: which conditions are associated with higher rates of non-participation in the labour force?

**DOI:** 10.1186/1471-2458-14-561

**Published:** 2014-06-05

**Authors:** Deborah Schofield, Michelle Cunich, Rupendra Shrestha, Megan Passey, Simon Kelly, Robert Tanton, Lennert Veerman

**Affiliations:** 1 NHMRC Clinical Trials Centre and School of Public Health, Sydney Medical School, The University of Sydney, Sydney, Australia; 2 University Centre for Rural Health – North Coast, School of Public Health, University of Sydney, Lismore, NSW, Australia; 3 National Centre for Social and Economic Modelling, University of Canberra, Canberra, ACT, Australia; 4 School of Population Health, University of Queensland, Brisbane, Queensland, Australia

**Keywords:** Chronic disease, Chronic disabling conditions, Labour force participation, Informal carers, Recipients of care, Household survey data, Cross-sectional study

## Abstract

**Background:**

Little is known about the effects of personal and other characteristics of care recipients on the behaviour of carers. The aim of this study is to examine the association between the main chronic (disabling) condition of care recipients and the likelihood of their (matched) primary carers aged 15–64 years being out of the labour force.

**Methods:**

We conducted a retrospective analysis of cross-sectional data from the Australian Bureau of Statistics 2009 Survey of Disability, Ageing and Carers (SDAC) for people aged 15–64 years. We estimated the rates of exit from the labour force for primary carers and non-carers; rates of chronic disease occurrence for care recipients living with their main carers; odds ratios of primary carers being out of the labour force associated with the main chronic condition of their care recipient who lives with them.

**Results:**

From the 2009 SDAC, we identified 1,268 out of 37,186 eligible participants who were primary carers of a care recipient who lived with them. Of these, 628 (49.5%) were out of the labour force. Most common diseases of care recipients were: back problems (12%); arthritis and related disorders (10%); diseases of the nervous system (such as multiple sclerosis, epilepsy, cerebral palsy) (7.4%); and conditions originating in the perinatal period or congenital malformations, deformations and chromosomal abnormalities (5.1%). When adjusted for age, sex, education and whether have a long term chronic condition of informal carers, the five conditions of care recipients associated with the highest odds of their carers being out of the labour force were: head injury/acquired brain damage; neoplasms, blood diseases, disorders of the immune system; leg/knee/foot/hip damage from injury/accident; dementia, Parkinson’s disease, Alzheimer’s disease; and diseases of the musculoskeletal system and connective tissue (osteoporosis).

**Conclusions:**

This study identifies the type of conditions that have the greatest impact on the labour force participation of informal carers – previously unavailable information for Australia. Australia, like most developed countries, is facing several skills shortages and an ageing population. These governments will need to adopt novel and more wholistic approaches to increase the labour force participation of diverse groups. Informal carers are one such group.

## Background

Globally, the number of children with a chronic illness or disability and older people needing care is projected to increase significantly in the next 20 years [1-3]. Focusing on older people, 13% of people aged 60 or older currently need long term care. However, the total number of older people with care needs is projected to almost treble from 101 to 277 million between 2010 and 2050 [4]. This care can be provided in both formal and informal ways: (a) formally by people employed in the health care sector, and (b) informally by relatives or friends of the recipient of care [5]. Informal carers are generally not paid for their caring responsibilities but these tasks can have an impact on the capability of carers to undertake paid employment [6]. In this paper, we focus on the impact of chronic illness or disability of care recipients on the labour force participation of informal caregivers.

Informal carers of people with a chronic illness or disability constituted 12% of the Australian population (or 2.6 million people) in 2009 [7], and this number is expected to increase due to greater demand for such care. In adults, greater demand is largely due to Australia’s ageing population [1] and related increasing rates of several chronic diseases [8], such as diabetes and dementia [9]. In children, the reasons for greater demand are more complex; there has been an increase in the rate of preterm births (babies born less than 37 gestational weeks) [10,11] and increases in the survival rates of all preterm babies, even extremely preterm babies (born less than 28 gestational weeks), with an associated increase in disability amongst those survivors [12]. The sizeable devotion of Australians to giving informal care has significant ramifications for national labour force participation, GDP, the tax base and welfare costs (and thus the budget balance), in addition to the individual financial, social, emotional and health costs of those carers.

Recognising the current (and projected) ‘crisis’ in informal care provision, there is a large body of literature on the impact of informal caregiving (full- or part-time basis) on labour force participation. These studies have found that informal carers tend to have lower rates of labour force participation compared to non-carers [6,13-16]. A recent cross-sectional study involving Australians aged 45 and older (n = 265,515) demonstrated that full-time carers are not only more likely to be out of the labour force than non-carers (and part-time carers) but more likely to have lower household income than non-carers. Just over 40% of non-carers had an annual household income of more than $70,000, whereas only 12.6% of full-time carers had the same level of income. These impacts were also found to be greater for carers who were themselves in poorer health [17]. A study using a single wave of the Household Income and Labour Dynamics in Australia (HILDA) survey data (2008) [18] confirms these prior findings. This study reports that being a main carer reduces the probability of employment by around 12 percentage points for both males and females, regardless of whether or not the carer lives with the recipient of care.

Gray and Edwards (2009), using a nationally representative sample of female carers in receipt of government assistance (2006 Families Caring for a Person with a Disability Survey), examined the impact of personal and care-related characteristics on the likelihood of maintaining employment [19]. They found that although carers had a lower employment rate than non-carers, over half of those who were not employed reported they would like to be in paid employment. The main factors associated with the lower rate of employment for female carers were having a low level of educational attainment, poorer health, caring for someone on a full time basis, caring for a child who has a disability, and not having someone outside the household who can provide assistance. Qu et al. (2012), using the 2009 Survey of Disability, Ageing and Carers (SDAC) and 2006 (Australian) Census data, examined the characteristics of parent carers and their son or daughter with a disability who lives with them [20]. With regard to economic outcomes, they found that older women (aged 65 years or older) caring for their adult children were less likely to participate in the labour force and had a lower personal income than others. Thus older women caring for a son or daughter with a disability (particularly female sole parents) were found to be disadvantaged in relation to financial provision for their own retirement.

Few studies have examined the impact of informal caregiving on the economic circumstances of carers (and their families) using Australian longitudinal data. Bittman et al. (2007) examined the effects of informal caregiving on carers’ employment, income and earnings using four waves of HILDA (2001–04) [21]. They found that carers were more likely to reduce their hours of work or exit from the labour force and earned less on average than non-carers. Leigh (2009) examined changes in labour market outcomes for carers as their caring commitments changed using a longer span of HILDA data (i.e. 2001–2007) [22] and found that caregiving had a negative effect on the labour force participation of carers. Falkiner (2011), using the latest waves of HILDA (waves 5–9 i.e. 2005–2009), examined the characteristics of people who become carers including the age at which people have the greatest risk of becoming carers for the first time [23]. This study provides estimates of the hazard of entering caregiving but it does not explore how this decision affects economic outcomes of carers (and families) after doing so.

Few studies, however, have examined the *type* of chronic conditions of care recipients that have the greatest impact on the labour force participation of informal carers, and even fewer on the economic consequences of health interventions which may, indirectly, improve the income of informal carers and government finances [8]. In this paper, we address this paucity in research in relation to the former. Specifically, we undertake a retrospective cross-sectional analysis (using the 2009 SDAC) to examine the associations between chronic (disabling) conditions of care recipients living with their primary (informal) carers and the likelihood of these carers of working age (i.e. aged 15–64 years) being out of the labour force.

## Methods

Data on people aged 15–64 years from the Australian Bureau of Statistics (ABS) 2009 Survey of Disability, Ageing and Carers (SDAC) [24] was used to identify the main chronic (disabling) condition of each (main) care recipient living with their matched primary informal carer(s). The use of these data was approved by the Australian Bureau of Statistics Microdata Review Panel.

### Identifying informal carers in the 2009 SDAC

There are several categories of carers in the 2009 SDAC. Carers were identified as those who indicated they were:

a) a primary carer of a usual resident (i.e. lives with) care recipient (care recipient identified and carer identified);

b) other primary carer of a usual resident care recipient;

c) primary carer of a non-usual resident (i.e. does not live with) care recipient; or

d) other carer.

However, information on both the main care recipient and carer (such as age, sex, education, labour force participation, chronic conditions, and duration of care) needed for this study was only available for main usual resident care recipients who could be matched to primary carers. In particular, information on the main disabling chronic condition of care recipients was only available for those who were living with their primary carers. For this reason, our analysis focuses only on primary carers of care recipients who live with them (i.e. categories (a) and (b) above).

A working definition of primary carers is provided by the ABS (2010) as follows: "A primary carer is a person who provides the most informal assistance, in terms of help or supervision, to a person with one or more disabilities or aged 60 years and over. The assistance has to be ongoing, or likely to be ongoing, for at least six months and be provided for one or more of the core activities (communication, mobility and self-care)" (p. 34).

The labour force was defined as people who are employed or unemployed but looking for work.

The 2009 SDAC contains information on the demographic and socioeconomic characteristics of all people surveyed (including age, sex, education, labour force participation) as well as their health and wellbeing (such as chronic conditions). For care recipients, there is detailed information on several aspects of their health and care arrangements, including the number and type of chronic (and disabling) conditions, number of carers they have, whether they are living with carer(s), and duration of care. This paper focuses only on the main disabling condition of care recipients for the analysis.

The survey data are weighted by the ABS to address the issue of unequal probability of selection in the survey, and to make the survey data a true representation of the Australian population. We used these weightings in our analysis to estimate the prevalence of chronic conditions of care recipients living with their carers for the entire Australian population needing care.

We used logistic regression analysis, adjusted for age and sex of the primary carer, to estimate the odds ratios (ORs) of them being out of the labour force associated with each (main) disabling condition of their care recipient who lives with them, using “not a carer” as the reference group. ORs are presented with 95% confidence intervals. Significance was set at P < 0.05. All analyses were conducted using SAS, version 9.3 (SAS Institute, Cary, NC, USA).

## Results

Of the 72,075 people surveyed in the 2009 SDAC, 37,186 (51.6%) were in the 15–64 years age group. Of these, 1,268 (3.4%) were identified as primary (informal) carers. Of the primary carers identified of working age, 628 (49.5%) stated they were caregiving and *out* of the labour force, with the remaining 640 (50.5%) stating they were caregiving and *in* the labour force (Table 1).

**Table 1:** People not in the labour force by carer status, age group and sex, of 37 186 Australians aged 15–64 years surveyed in 2009

	**No. not in labour force in survey group**											
**Age group (years)**	**Men**				**Women**				**All**			
	Primary carers*		**Not carers**		Primary carers*		**Not carers**		Primary carers*		**Not carers**	
	**n**	**Wt n (%)**^**^**^	**n**	**Wt n (%)**^**^**^	**n**	**Wt n (%)**^**^**^	**n**	**Wt n (%)**^**^**^	**n**	**Wt n (%)**^**^**^	**n**	**Wt n (%)**^**^**^
15-24	6	2 536 (34.3)	1 083	391 330 (27.7)	20	5 842 (54.4)	1 061	379 885 (28.3)	26	8 378 (46.2)	1515	771 215 (28.0)
25–34	3	1 802 (18.6)	222	88 338 (6.2)	55	17 947 (43.5)	810	289 033 (21.5)	58	19 749 (38.8)	629	377 371 (13.6)
35–44	23	9 492 (37.8)	228	79 501 (5.9)	115	40 824 (46.6)	810	268 616 (21.1)	138	50 316 (44.7)	505	348 117 (13.3)
45–54	43	14 606 (30.4)	312	103 789 (8.5)	109	37 531 (46.1)	603	200 817 (17.5)	152	52 137 (44.7)	527	304 606 (12.8)
55-64	83	26 783 (51.6)	731	242 927 (24.8)	172	57 790 (70.1)	1 261	408 195 (44.5)	254	84 573 (62.9)	575	651 122 (34.3)
Total	158 (42.8)	55 219 (42.6)	2 576 (14.2)	905 885 (14.2)	471 (52.4)	159 934 (52.7)	4 545 (25.5)	1 546 546 (25.6)	628 (49.5)	215 153 (49.7)	7 121 (19.8)	2 452 431(19.7)

n = survey sample.

Wt n = weighted sample (reflect Australian population).

*Primary carers = Primary carers of usual resident (i.e. lives with) care recipients.

^The proportion of each cohort not in the labour force.

All primary carers identified could be matched to a main care recipient who lived with them - information needed to identify the main disabling condition of care recipients. The most common disabling conditions among care recipients were: back problems, arthritis and related disorders, diseases of the nervous system (such as multiple sclerosis, epilepsy, and cerebral palsy), autism and related disorders, and congenital conditions (Table 2). Based on the proportion of primary carers out of the labour force, the most work-limiting conditions for caregivers were: head injury/acquired brain damage, schizophrenia, diseases of the musculoskeletal system and connective tissue (osteoporosis), dementia/Parkinson’s disease/Alzheimer’s disease, and endocrine/nutritional and metabolic disorders (thyroid, diabetes and high blood pressure) (Table 2). 60-70% of carers who were caring for someone with one of these conditions were not in the labour force.

**Table 2:** Prevalence of main disabling conditions of care recipients living with carers and the labour force participation of primary carers among 37 186 Australians aged 15–64 years, 2009

**Main disabling condition of care recipient**	**Total**		**Not in labour force**	
	**No. in survey**	**Weighted number (%)**	**No. in survey**	**Weighted number (%)**
Not a carer	35 918	12 427 469 (96.6)	7 121	2 456 120 (19.8)
Primary carer of UR recipient of care	1 268	433 338 (3.4)	628	215 477 (49.7)
Main disabling condition of UR recipient of care:				
Neoplasms (tumours/cancers), blood diseases, disorders of immune system	33	10 535 (3.4)	19	6 392 (57.6)
Endocrine/nutritional and metabolic disorders (thyroid, diabetes, high blood pressure)	15	5 275 (1.2)	9	3 234 (60.0)
Schizophrenia	13	4 776 (1.1)	8	2 792 (61.5)
Depression/mood affective disorders	38	13 401 (3.1)	20	6 354 (52.6)
Phobic and anxiety disorders, nervous tension/stress	39	13 439 (3.1)	15	5 686 (38.5)
Intellectual and developmental disorders, mental retardation/intellectual disabilities	62	18 943 (4.4)	34	10 300 (54.8)
Autism and related disorders (including Rett's syndrome and Asperger's syndrome)	88	26 693 (6.2)	36	11 210 (40.9)
ADD/hyperactivity	28	9 195 (2.1)	11	2 955 (39.3)
Other mental and behavioural disorders	59	19 756 (4.6)	29	9 905 (49.2)
Dementia, Parkinson’s disease, Alzheimer’s disease	29	9 644 (2.2)	18	6 037 (60.1)
Other diseases of the nervous system (MS, epilepsy, cerebral palsy, paralysis, chronic fatigue syndrome)	87	32 070 (7.4)	34	12 157 (39.1)
Diseases of the eye and adnexa (retinal disorders/defects, glaucoma, sight loss)	22	9 539 (2.2)	9	4 215 (40.9)
Diseases of the ear and mastoid process (deafness/hearing loss congenital)	44	14 330 (3.3)	21	6 827 (47.7)
Heart diseases	28	9 133 (2.1)	15	4 666 (53.6)
Diseases of the circulatory system (stroke and hypertension)	51	18 058 (4.2)	28	10 380 (54.9)
Diseases of the respiratory system (emphysema, asthma)	39	13 568 (3.1)	20	7 401 (51.3)
Arthritis and related disorders	123	43 291 (10.0)	57	19 364 (46.3)
Back problems (dorsopathies)	153	51 972 (12.0)	82	28 709 (53.6)
Other soft tissue/muscle disorders (including Rheumatism)	15	6 089 (1.4)	7	2 452 (46.7)
Diseases of the musculoskeletal system and connective tissue (osteoporosis)	31	11 115 (2.6)	19	6 534 (61.3)
Conditions originating in perinatal period or congenital malformations, deformations and chromosomal abnormalities	59	22 006 (5.1)	25	10 110 (42.4)
Head injury/acquired brain damage	42	13 409 (3.1)	28	9 842 (66.7)
Leg/knee/foot/hip damage from injury/accident	46	14 665 (3.4)	26	8 628 (56.5)
Other damage from injury/accident (arm/hand/shoulder damage)	24	7 447 (1.7)	8	2 299 (33.3)
Other long term condition	100	34 988 (8.1)	50	17 028 (50.0)

Crude ORs (not shown) revealed a significant association with primary carers being out of the labour force for all chronic (disabling) conditions of care recipients, except for Attention Deficit Disorder/hyperactivity, arm/hand/shoulder damage from injury or accident, and other soft tissue/muscle disorders. After adjusting for the carer’s age, sex, education and whether they have a chronic condition themselves, all associations apart from schizophrenia and the three condition groups noted above remained significant (Table 3).

**Table 3:** Main disabling conditions of care recipients living with carers associated with carers being out of the labour force

**Condition**	Adjusted OR*	**95% CI**	
Neoplasms (tumours/cancers), blood diseases, disorders of immune system	6.547	2.844	15.076
Endocrine/nutritional and metabolic disorders (thyroid, diabetes, high blood pressure)	4.239	1.303	13.787
Schizophrenia	3.933	0.709	21.817
Depression/mood affective disorders	2.249	1.110	4.557
Phobic and anxiety disorders, nervous tension/stress	2.398	1.100	5.231
Intellectual and developmental disorders, mental retardation/intellectual disabilities	4.373	2.494	7.666
Autism and related disorders (including Rett's syndrome and Asperger's syndrome)	3.227	1.906	5.462
ADD/hyperactivity	1.429	0.633	3.225
Other mental and behavioural disorders	3.467	1.953	6.155
Dementia, Parkinson’s disease, Alzheimer’s disease	4.932	1.845	13.186
Other diseases of the nervous system (MS, epilepsy, cerebral palsy, paralysis, chronic fatigue syndrome)	2.527	1.569	4.071
Diseases of the eye and adnexa (retinal disorders/defects, glaucoma, sight loss)	2.716	1.078	6.845
Diseases of the ear and mastoid process (deafness/hearing loss congenital)	2.704	1.307	5.593
Heart diseases	3.605	1.651	7.872
Diseases of the circulatory system (stroke and hypertension)	3.759	1.919	7.363
Diseases of the respiratory system (emphysema, asthma)	3.402	1.565	7.391
Arthritis and related disorders	2.488	1.628	3.803
Back problems (dorsopathies)	4.346	2.945	6.414
Other soft tissue/muscle disorders (including Rheumatism)	2.385	0.849	6.695
Diseases of the musculoskeletal system and connective tissue (osteoporosis)	4.606	2.134	9.941
Conditions originating in perinatal period or congenital malformations, deformations and chromosomal abnormalities	2.605	1.434	4.732
Head injury/acquired brain damage	8.415	4.137	17.116
Leg/knee/foot/hip damage from injury/accident	5.781	2.919	11.451
Other damage from injury/accident (arm/hand/shoulder damage)	1.062	0.400	2.816
Other long term condition	0.529	0.497	0.564

OR = odds ratio. *Adjusted for age, sex, education of primary carer as well as whether they have a long term health condition. The reference group was “non-carer”.

## Discussion

Using population data from the ABS, we estimated that 215,153 Australians of working age were missing from the labour force and giving care to a relative or friend living with them who had a chronic (disabling) condition in 2009. Back problems, arthritis and related disorders, diseases of the nervous system (such as multiple sclerosis, epilepsy, and cerebral palsy), mental and behavioural disorders (autism, congenital conditions, and intellectual and developmental disorders/mental retardation/intellectual disabilities) and diseases of the circulatory system (stroke and hypertension) were the five most common conditions among those care recipients and accounted for 47% of total caregiving. This profile is similar to the profile of disorders accounting for most Disability Support Pension payments. Musculoskeletal disorders, psychological problems, and diseases of the circulatory system are the top three long term conditions reported by recipients of the disability pension (Centrelink, Performance and Information Branch, data request BI3268: health conditions associated with sickness benefits and Disability Support Pension, 13 Jan 2006). The five diseases of care recipients associated with the highest odds of carers being out of the labour force were: head injury/acquired brain damage, schizophrenia, diseases of the musculoskeletal system, dementia/Parkinson’s disease/Alzheimer’s disease, and endocrine/nutritional and metabolic disorders. 60-70% of primary carers who were out of the labour force were caring for someone with one of these conditions.

The study has a number of limitations. Firstly, the impact of chronic conditions of care recipients on the labour force participation of carers relies on self-reported data on the care recipient’s main disabling condition. Although self-assessed health is regarded as a valid measure of health status [25], there is possible bias in the results as not all self-reported conditions from the 2009 SDAC (or similar surveys) have been appraised in terms of reliability/validity. Secondly, as the 2009 SDAC are cross-sectional data, it is only possible to identify associations rather than causal relationships between variables. Finally, the 2009 SDAC does not capture mortality data, and therefore the study could not estimate the impact of mortality (care recipient or carer) on labour force participation.

Previous governments have focused on increasing labour force participation via economic incentives targeting younger and older workers separately. For new parents, there have been improvements in parental leave allowances for women and men in all workplaces, as well as commencement of the Commonwealth Government’s paid parental leave which provides payments for up to 18 weeks after the birth or adoption of a child [26]. For older workers, the 15% tax on lump sums and pensions from superannuation schemes after the age of 60 years has been removed [27]. There is also the Age Discrimination Act 2004 which provides job protection for all Australian workers [28]. However, these broad-reaching incentives to help people either return to, or remain in, paid employment fail to take into account one of the main reasons people have to leave the labour force abruptly: the caring needs of a relative or friend. Moreover, there needs to be more achieved in terms of policy design – we need to address the rise of chronic conditions which are associated with most of the low labour force participation of carers. Until then, these incentives are unlikely to have a major impact on the labour force participation of caregivers.

Traditionally, health policy has focused on the provision of health care (and services) to improve the health of citizens for its own sake, and employment policy (and setting of priorities) has been largely conducted in isolation from health policy. However, recent Australian Government health reforms seem to echo the notion that “good health policy is part of good economic policy” as noted in Russell et al. (2008) [29]. This philosophy naturally leads to the need to address the country’s increasing burden of preventable illnesses/diseases, such as diabetes and cardiovascular disorders, with the highest care demands, and reduce the occurrence of accidents and injuries.

The challenges faced by carers as a whole seem to be from pressures already in the health sector and the lack of formal measures ensuring appropriate workplace flexibility for carers. Whilst there are public and private care services for those with a disability, chronic condition, or frail aged (such as residential and aged care facilities) and respite care (see, for details about the Government’s National Respite for Carers Program, [30]), there are more people in need of these services than is available, resulting in delayed or constrained access [31]. Moreover, the policy direction taken in the last 20 years has been to move away from institutional forms of care to “ageing in place” i.e. community-based care [32].

The Australian Government provides two forms of financial supports for carers: Carer Payment and Carer Allowance. The former is an income support payment for people who personally provide regular (continuous) care in the home for someone with a severe disability, illness, or frail aged. It is a payment for carers who are unable to participate in the labour force full time due to their caregiving role. Eligibility for this payment includes satisfying an income and assets test. It also depends on the level of impairment of the care recipient [19,33]. The latter is a supplementary payment for carers who provide additional daily care and attention for someone with a disability or medical condition, or frail aged [33]. Eligibility for this payment does not include income or asset testing. Almost everyone who receives Carer Payment also receives Carer Allowance. Currently, the single rate for Carer Payment is $766.00 per fortnight and the couple rate for this payment is $577.40 per fortnight. Carer Allowance is currently $118.20 per fortnight. Additionally, an annual payment of $600 is payable for each child cared for under the age of 16 years [33].

There is limited information on the impact of receiving carer payments on carers’ labour force participation and employment. Gray and Edwards (2009) examined the labour force participation of female carers, taking into account whether they were receiving carer payments or not [19]. (Leigh (2010)[22] only uses receipt of government payments for carers to identify carers in HILDA). Gray and Edwards (2009) reported the employment rate of female carers who received Carer Allowance only to be 43.1% (11% of whom were in full time employment) whereas the employment rate of carers who received Carer Payment only was 20.5% (less than 1% were in full time employment). Although these relationships were significant from the estimated regression models, there was no significant relationship between type of carer’s payment received and the desire to commence work. Availability, access to, and quality of suitable care services and supports for carers (such as financial supports, and having someone to help provide care at short notice) can affect working carers’ chances of being able to integrate work and care successfully. Information on the type and amount of government assistance received by carers (i.e. the exact amount of Carer Payment and Carer Allowance) is not available in the 2009 SDAC; however, how this impacts on work and caregiving decisions is an issue requiring further research.

Employment policy in Australia and whether working carers (of adult people) have suitable forms of support available in work life may also need further attention. Working parents and carers are (legally) protected from discrimination when attempting to balance their work schedules with family and caring responsibilities. Under the Equal Opportunity Act 2010, employers have a positive responsibility to take practical and comparable measures to remove discrimination, sexual harassment and victimisation from their workplace (such as bullying and intimidation by other employees; an employee being denied a promotion or being moved to a position with lower responsibility; dismissal from employment; an employee being denied additional contract work) as much as possible. The Act applies to employers (organisations) of all sizes, includes all types of workers, and is relevant to all stages of employment. Thus there are legal protections in place to enable carers to manage their work and caring responsibilities effectively [34]. Thus the challenges seem to be in relation to whether (a) workers feel they are able to discuss any difficulties they are facing with employers at the point they occur, and (b) employers are able (and it is financially viable to do so) to generate the degree of workplace flexibility (flexible work hours, part-time work or paid/unpaid care leave hours) required to meet the personal needs of their workers who are also carers.

While 54% of all carers are women [35], recent studies have shown that men are taking on caring roles more often than was previously assumed, with a larger number of men being primary carers than women in the age group 65 years or older in Australia [36] which is driven, in part, by the longer life expectancy of women and the increasing prevalence of people with dementia and other cognitive disorders [37]. As noted in this study, caregiving has negative effects such as reduced labour force participation and lower household income among all carers. However, previous studies have also demonstrated that male and female carers differ in the way they seek to manage work and caregiving. Women more often reduce their hours of work or relinquish work when caregiving [36] and thus female carers experience additional risks in terms of career development and income compared to male carers [17,21]. This study provides support for the Australian Government’s current health proposals which include improving the opportunities for working-age carers to participate in the labour force at a desired level [38]. With persistent skills shortages and an ageing population requiring more care in the future, the Government will need to continue on its path of adopting a more targeted (but wholistic) approach to increasing the labour force participation of its working-age population. Special attention will need to be given to the challenges faced by important subgroups. Informal carers are one such group.

## Conclusions

This study identifies the type of conditions that have the greatest impact on the labour force participation of carers – information previously unavailable. Given the challenges facing Australia and other developed countries (such as severe skills shortages, an ageing population, rising health care costs), successive governments will need to adopt fresh and more wholistic approaches to increase the labour force participation of different subgroups, including carers.

## Competing interests

This study is part of continuing research funded by a National Health and Medical Research Council (NHMRC) Partnership Project (APP 1055037), Pfizer Australia and Carers Australia. All authors are independent from the funding sources.

## Authors’ contributions

DS conceived the study, and contributed to its design and coordination. DS also led drafting of the manuscript. MC contributed to the design of the study, drafting the manuscript, and performed the statistical analysis. RS contributed to the design of the study, and provided expert advice on statistical modelling. MP and LV contributed to the design of the study, and provided expert advice on the long- and short-impacts of chronic conditions. SK and RT contributed to the design of the study, and provided expert advice on carers. All authors contributed to interpretation of the results. All authors read and approved the final manuscript.

## Pre-publication history

The pre-publication history for this paper can be accessed here:

http://www.biomedcentral.com/1471-2458/14/561/prepub
